# Sustained remission following the discontinuation of tofacitinib in patients with rheumatoid arthritis (XANADU study): an open-label randomised study

**DOI:** 10.1136/rmdopen-2023-003029

**Published:** 2023-04-25

**Authors:** Satoshi Kubo, Yusuke Miyazaki, Koichi Amano, Kiyoshi Matsui, Hideto Kameda, Yoshino Inoue, Shingo Nakayamada, Takehisa Ogura, Yuko Kaneko, Kunihiro Yamaoka, Yoshiya Tanaka

**Affiliations:** 1 Department of Molecular Targeted Therapies, University of Occupational and Environmental Health Japan, Kitakyushu, Japan; 2 The First Department of Internal Medicine, University of Occupational and Environmental Health Japan, Kitakyushu, Japan; 3 Department of Rheumatology and Clinical Immunology, Saitama Medical Centre, Saitama Medical University, Saitama, Japan; 4 Division of Allergology and Rheumatology, Department of Diabetes Endocrinology and Clinical Immunology, Hyogo Medical University, Hyogo, Japan; 5 Division of Rheumatology, Department of Internal Medicine, Toho University Ohashi Medical Center, Tokyo, Japan; 6 Division of Rheumatology, Department of Internal Medicine, Keio University School of Medicine, Tokyo, Japan; 7 Department of Rheumatology and Infectious Diseases, Kitasato University School of Medicine, Sagamihara, Japan

**Keywords:** arthritis, rheumatoid, therapeutics, antirheumatic agents

## Abstract

**Objective:**

To investigate sustained remission following the discontinuation of tofacitinib in patients with rheumatoid arthritis.

**Methods:**

Patients who had an inadequate response to methotrexate (MTX) with or without biological disease-modifying antirheumatic drugs were randomly divided into two groups at baseline, and tofacitinib treatment in combination with MTX was administered to both groups. Either MTX or tofacitinib was then withdrawn if patients achieved Clinical Disease Activity Index remission at week 52. The primary outcome was the proportion of patients who sustained clinical remission at week 104.

**Results:**

A total of 113 patients participated in this study. Among them, a total of 48 patients achieved remission at week 52. After discontinuation of tofacitinib, only 29.2% (7/24) of patients remained remission, while 50.0% (10/20) of patients, which was numerically higher but not statistically significant, sustained remission after MTX discontinuation. A greater proportion of bio-naïve patients achieved remission at week 52 and sustained low disease activity with tofacitinib discontinuation at week 104. Additionally, the patients who were able to discontinue tofacitinib without flares had lower rheumatoid factor (p=0.04) and lower anti-cyclic citrullinated peptide antibody (p=0.051) before discontinuation of tofacitinib. No severe adverse events were recorded after discontinuation of tofacitinib or MTX. In patients who relapsed after tofacitinib discontinuation, 71.4% achieved remission with resumption of tofacitinib.

**Conclusions:**

This study implies that a blanket cessation of tofacitinib may not be suitable for all patients, given that 58% of the participants experienced relapse. However, the withdrawal of tofacitinib is unlikely to result in the acquisition of treatment-resistance.

**WHAT IS ALREADY KNOWN ON THIS TOPIC**

- Discontinuation of certain classes of biological disease-modifying antirheumatic drugs (bDMARDs) following clinical remission can be successful. However, there is also persuasive evidence indicating that discontinuing bDMARDs may eventually result in flares in the majority of patients. Randomised control studies evaluating the discontinuation of targeted synthetic DMARDs are lacking.

**WHAT THIS STUDY ADDS**

- This is an open-label multicentre, prospective and randomised controlled study aimed to evaluate the discontinuation of tofacitinib, a pan Janus kinase (JAK) inhibitor in patients who had achieved clinical remission, with a comparison of discontinuation of methotrexate (MTX). Approximately 60% of patients who discontinued tofacitinib relapsed, whereas 35% of patients relapsed after MTX discontinuation. The majority of patients who flare can again achieve remission following re-administration of tofacitinib.

**HOW THIS STUDY MIGHT AFFECT RESEARCH, PRACTICE OR POLICY**

- This study showed that the discontinuation of JAK inhibitors would be difficult. However, it is possible if patients are appropriately selected. Specifically, low titre seropositivity can contribute to the success of drug withdrawal. This might lead to a reduction in healthcare economic burden and risk of adverse effects.

## Introduction

With the development and launch of the Janus kinase (JAK) inhibitor, tofacitinib, treatment options for rheumatoid arthritis (RA) have expanded, which has enabled more patients to achieve clinical remission.^1^ Following the success of tofacitinib, a number of JAK inhibitors have been developed and applied to other autoimmune diseases besides RA.^2^ However, there are some concerns about JAK inhibitors: for example, the occurrence of adverse events such as lymphopenia, reactivation of herpes zoster virus and hyperlipidemia complicates the continued use of JAK inhibitors in some patients.^3–5^ In addition, a long-term clinical study of tofacitinib has reported the incidence of major adverse cardiovascular events and malignancies as well as a potential increase in the risk of deep vein thrombosis.^6–8^ Given that of the different JAK inhibitors, the most abundant clinical data have been obtained for tofacitinib, such risks are likely to be shared among the remaining JAK inhibitors once more data have been collected. In response, the US Food and Drug Administration and European Medicines Agency have issued an alert on the risks of developing cardiovascular events and cancers due to JAK inhibitors.^9 10^ Therefore, it is valuable for shared decision making to clarify the probability of tofacitinib withdrawal without a relapse in disease activity in patients who achieved clinical remission under treatment with tofacitinib and methotrexate (MTX).

Achieving clinical remission in patients with RA is the primary goal and using disease-modifying antirheumatic drug (DMARDs) is essential.^11^ In the meantime, not only targeted synthetic DMARDs (tsDMARDs), but also other DMARDs could cause adverse events. The anchor drug for RA, MTX, also shows several adverse effects such as digestive symptoms, opportunistic infections and lymphoproliferative disorder.^12^ It is known that many patients want to taper MTX.^13^ Therefore, investigating the efficacy of sustained remission after discontinuation of DMARDs is an important area of study.

There are two major benefits to consider when deciding whether or not to withdraw tsDMARDs. One is from the perspective of adverse events as mentioned above: drug withdrawal can eliminate the risk of tsDMARD-related adverse events. The other is reducing medical costs. Since the costs of tsDMARDs are expensive, their withdrawal can mitigate the financial burden on patients as well as on society. Considering these factors, withdrawal of tsDMARDs is reasonable as an option for patients in sustained remission.^11^

We have previously reported the impact on disease activity after withdrawal of tofacitinib in patients who completed phase III and long-term extension study of tofacitinib.^14^ This non-randomised observational study suggested the possible maintenance of low disease activity after the withdrawal of a JAK inhibitor and the possible contribution of low titers of rheumatoid factor (RF) and anti-cyclic citrullinated peptide antibody (ACPA) levels to the sustained drug withdrawal. However, there is little evidence on the withdrawal of JAK inhibitors, and no evidence is available from randomised, controlled, prospective trials. Thus, we conducted this study to investigate sustained remission following the discontinuation of tofacitinib using the withdrawal of MTX as a control.

## Methods

### Patients

Patients with RA who had inadequate responses to MTX treatment with or without biological DMARDs (bDMARDs) were enrolled in this study. The detailed eligibility criteria are shown below (ineligibility criteria are described in the online supplemental information).

10.1136/rmdopen-2023-003029.supp1 Supplementary data

1. Subjects over 17 years of age.
2. Subjects willing and able to sign informed consent.
3. Subjects fulfilled either the 1987 revised criteria of the American Rheumatism Association or the 2010 classification criteria of the American College of Rheumatology (ACR)/EULAR.^15–17^
4. Inadequate response to treatment with oral MTX with or without bDMARDs for at least 8 weeks prior to the first dose of tofacitinib at a dose of 4–16 mg/week.
5. Subjects who have low to high disease activity. Disease activity is defined by Clinical Disease Activity Index (CDAI).
6. Subjects must have discontinued all bDMARDs prior to the first dose of tofacitinib. The washout period for bDMARDs (note: rituximab is not approved for the treatment of RA in Japan) is specified as follows: ≥8 weeks for infliximab, ≥2 weeks for etanercept and ≥4 weeks for other bDMARDs.
7. Subjects must have discontinued non-MTX conventional synthetic disease-modifying antirheumatic drugs (csDMARDs) (sulfasalazine, hydroxychloroquine, chloroquine and leflunomide) at least 4 weeks prior to the first dose of tofacitinib.

### Disease activity assessment

RA disease activity was defined by the CDAI.^18 19^ CDAI scores of 22.1–76.0 have been labelled as high activity, 10.1–22.0 as moderate activity, 2.9–10.0 as low activity and 0.0–2.8 as remission.

### Study design

The detailed study protocol is described in the online supplemental information. The XANADU study (XeljANz treatment for RA Aiming DrUg free) was conducted as a multicentre, prospective, open-label and randomised control study. A flow chart of the overall study is shown in online supplemental figure 1. All patients inadequately responded to background MTX with or without bDMARDs and were randomly assigned to one of two groups: the tofacitinib discontinuation group and the MTX discontinuation group at baseline. After randomisation, tofacitinib was administered in addition to MTX in all the patients of both groups. The results of the randomisation remained hidden from both patients and the investigators until week 52. At week 52, either tofacitinib or MTX was discontinued according to the assignments if a patient achieved sustained clinical remission as defined by CDAI. Specifically, sustained clinical remission was defined by the presence of remission at three consecutive visits. Patients who did not reach clinical remission at week 52 did not move to the next step. Patients from both groups were allowed to visit the outpatient clinic every month and had to visit for the periodical follow-up (weeks 56, 64, 78 and 90) to check for disease activity until week 104. Tender joint counts, swelling joint counts, patients’ global assessment, evaluator’s global assessment, C reactive protein and erythrocyte sedimentation rate were recorded. Disease flares are defined based on moderate or high disease activity, according to CDAI. Patients with flares in the tofacitinib or MTX discontinuation groups were re-administered tofacitinib or MTX, respectively. After week 52, in patients who progress to moderate disease activity, the observed disease activity at that time is carried forward to week 104. All serious adverse events (SAEs) leading to discontinuation of the drugs or withdrawal from the study were recorded throughout the study.

10.1136/rmdopen-2023-003029.supp2 Supplementary data

### Randomisation and masking

All participants were randomly assigned to one of two groups: the tofacitinib discontinuation group and the MTX discontinuation group at baseline. Randomisation was done on a 1:1 basis, and randomisation with assignment concealment is done by the central coordinator by use of a computer-generated simple randomisation method. After randomisation, either a dosage of tofacitinib 5 mg two times a day or 5 mg per day was administered in addition to MTX in both groups. The randomisation was performed at baseline but not at week 52 to facilitate the study procedures after week 52. The results of the randomisation remained hidden from both patients and the investigators until week 52. At week 52, either tofacitinib or MTX was discontinued according to the assignments if a patient achieved clinical remission as defined by CDAI. After discontinuation of the drug at week 52, patients did not take placebo pills, resulting in no blinding.

### Study endpoints

The primary endpoint of the XANADU study was the proportion of patients who sustained clinical remission at week 104. As outlined in the Study design section, subjects who experienced flares, which were defined as moderate or high disease activity based on the CDAI, were re-administered either tofacitinib or MTX, and these individuals were not considered to have met the endpoint even if they regained clinical remission. Short-term low disease activity was considered as sustained remission if remission was reached again without additional treatment. The secondary endpoints were the proportion of patients who sustained low disease activity at week 104, the proportion of patients who had SAEs throughout the study, and the proportion of patients who achieved clinical remission after rescue by re-administration of tofacitinib or MTX. Subgroup exploratory analyses were performed to reveal which patients could achieve successful drug discontinuation.

### Investigator sites and study period

The following facilities/hospitals participated in this study: University of Occupational and Environmental Health, Japan, Saitama Medical University, Hyogo Medical University School of Medicine, Toho University, Keio University School of Medicine and Kitasato University School of Medicine. This study was conducted between May 2014 and April 2022 (subject incorporation started on 27 August 2014, and the last case was incorporated on 20 February 2020).

### Statistical analysis

The data analysis plan is described in the online supplemental information. Briefly, the study was powered to detect a 10% difference in sustained remission rates at week 104 between the tofacitinib and MTX discontinuation groups. Assuming a sustained remission rate of 10% in the tofacitinib discontinuation group and 20% in the MTX discontinuation group, a total sample size of 110 patients, would provide 80% or more statistical power to detect the difference between the two treatment groups with a two-sided 5% significance level, allowing for an approximate dropout rate of 5%.

The baseline demographics and clinical characteristics of general practice clusters and individual participants were reported. For the continuous variables (eg, age), either mean and SD was presented or median and IQR depending on the distribution of the data. The number of observations used in each calculation was shown alongside the summaries. For the categorical variables (eg, sex), the number and percentage of participants in each of the categories and the total number of observations were presented. A comparison of the responses, overall scoring and subgroup numbers was undertaken. All baseline summaries were presented and reported for each intervention arm and in total. Consolidated standards of reporting trials (CONSORT) guidelines generally do not recommend statistical significance testing of baseline imbalances between the arms.

The primary outcome was analysed using a χ^2^ test with a two-sided 5% significance level. Other DMARDs were used in patients who could not achieve clinical remission or low disease activity before week 52. In these subjects, an intention-to-treat analysis was used, and the observed disease activity at the time of other DMARDs administration was carried forward to week 52. After week 52 (drug discontinuation), patients who progressed to moderate disease activity (CDAI >10.0) were re-administered tofacitinib or MTX, and the observed disease activity at that time was carried forward to week 104.

Similar to the analysis of the primary outcome, the secondary outcomes were analysed using a χ^2^ test with a two-sided 5% significance level.

Subgroup exploratory analysis to identify the prognostic factors for successful withdrawal for the primary and secondary outcomes was conducted using univariate analysis. The optimal cut-off value for the prognostic factor was calculated using receiver operator characteristic (ROC) curve analysis. All analyses were performed using JMP V.11.0 (SAS Institute).

## Results

### Baseline characteristics

One hundred and thirteen patients who had inadequate responses to MTX (MTX-IR) with or without bDMARDs were enrolled in this study. The overall design, including patient inclusion and exclusion criteria of the study, was described in online supplemental information and online supplemental figure 1. In summary, 113 patients were first randomised into two strategies: 56 patients in the discontinuation of tofacitinib and 57 patients in the discontinuation of MTX. All patients were then treated with tofacitinib in combination with MTX. The results of the randomisation were blinded to both the investigator and the patients until week 52. The background of the patients in each group is shown in table 1. The mean age of the patients was 58 for those in the discontinuation of the tofacitinib group and 57 years for those in the discontinuation of the MTX group. Seventy-one per cent and 63% of the patients in each respective group had failed at least one biological agent. The mean disease activity at baseline was 23.9 and 22.9 for CDAI, respectively. The baseline characteristics of the two groups were statistically comparable except for RF and ACPA positivity. This accidental bias, which was triggered by simple randomisation, may have potentially impacted the results of this study, especially in the comparison between tofacitinib discontinuation and MTX discontinuation.

**Table 1:** Baseline demographic and clinical characteristics of the included patients

Variables	Discontinuation of tofacitinib (n=56)	Discontinuation of methotrexate (n=57)
Age (years)	56.6 (14.1)	60.1 (11.8)
Gender, n (% female)	48 (85.7)	47 (82.5)
Disease duration (month)	96.6 (99.7)	110.9 (103.3)
Steinbrocker’s classification, %, I/II/III/IV	18/52/12/18	25/40/16/19
Treatment history		
Prior use of biologics	40 (71.4%)	36 (63.2%)
MTX use at baseline, n (%)	56 (100.0)	57 (100.0)
Dose, mg/week	12.4 (3.5)	11.4 (4.0)
Glucocorticoid use at baseline, n (%)	6 (10.7)	4 (7.0)
Dose, mg/day	4.4 (1.0)	5.0 (2.0)
Tofacitinib dose/day, n (%)	5 mg; 2 (3.6), 10 mg; 54 (96.4)	5 mg; 6 (10.5), 10 mg; 51 (89.5)
28-Tender joint count	8.4 (6.0)	8.8 (5.8)
28-Swollen joint count	8.2 (4.7)	7.0 (4.5)
PGA, VAS 0–100 mm	55.6 (25.3)	48.7 (22.6)
EGA, VAS 0–100 mm	46.3 (21.8)	44.0 (21.2)
DAS28-ESR	5.5 (1.3)	5.2 (1.2)
CDAI	27.4 (12.2)	25.5 (11.2)
HAQ-DI	1.3 (0.8)	1.2 (0.8)
CRP (mg/dL)	2.1 (3.3)	1.9 (3.1)
ESR (mm/hour)	45.4 (32.6)	39.0 (30.5)
Rheumatoid factor (U/mL)	141.9 (168.9)	173.1 (355.6)
Positive (%)	50 (89.3)	36 (63.2)
Anti-CCP antibody (U/mL)	186.7 (291.9)	179.1 (411.0)
Positive (%)	49 (87.5)	39 (68.4)
MMP-3 (ng/mL)	264.4 (383.3)	198.2 (269.8)

Data reported as mean (SD).

CCP, cyclic citrullinated peptide; CDAI, Clinical Disease Activity Index; CRP, C reactive protein; DAS28, Disease Activity Score 28 Joint; EGA, Evaluator’s Global Assessment; ESR, erythrocyte sedimentation rate; HAQ, Health Assessment Questionnaire; MMP-3, matrix metalloproteinase-3; MTX, methotrexate; PGA, Patient’s Global Assessment.

### Achievement of successful drug withdrawal at week 104

**Figure 1:**
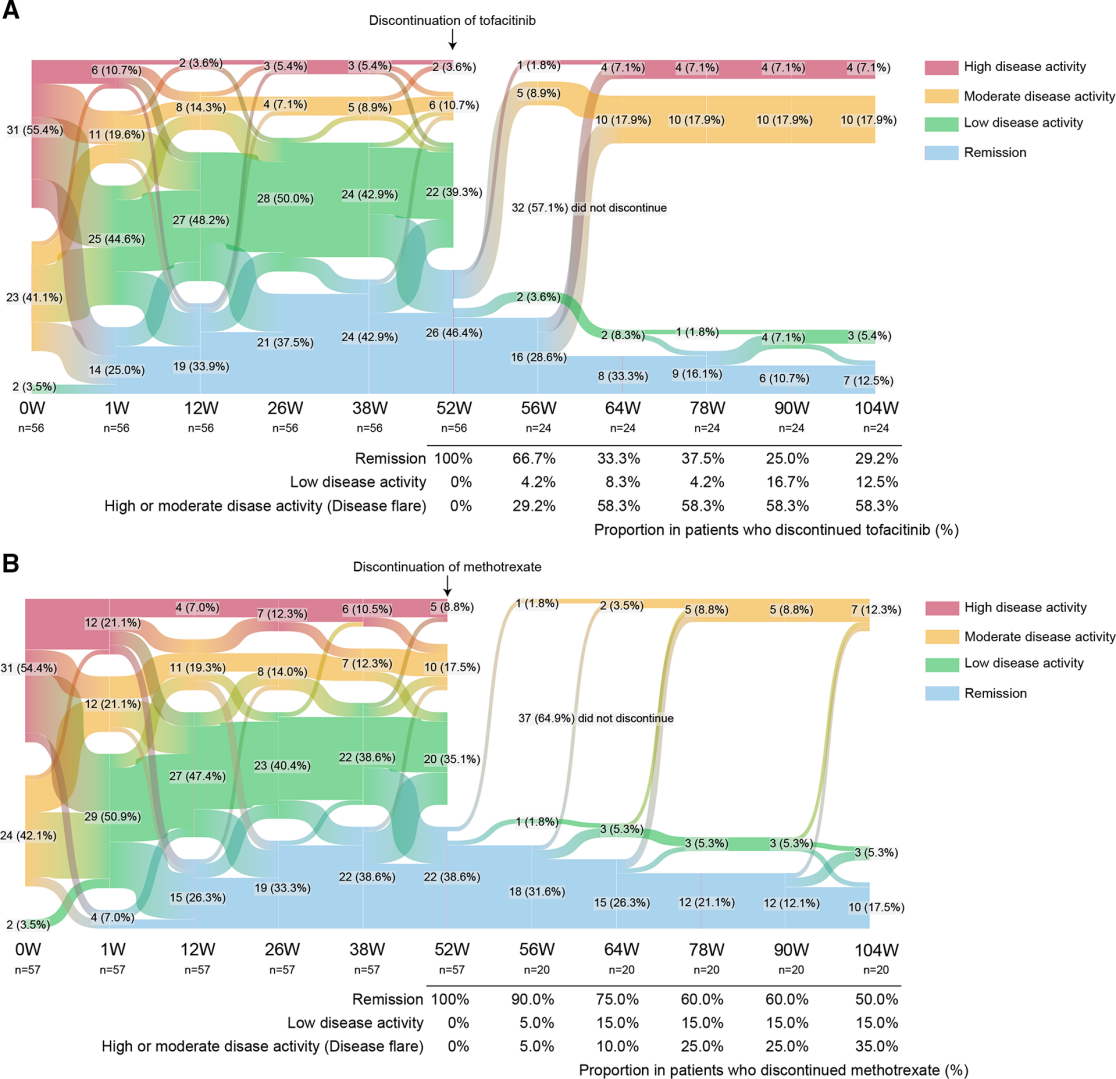
Sankey diagram describing proportion of patients per CDAI disease activity category over time. (A) Tofacitinib discontinuation arm (n=56), (B) methotrexate discontinuation arm (n=57). Data are n (%). CDAI, Clinical Disease Activity Index.

The changes in CDAI recorded over 104 weeks are shown in figure 1. The mean CDAI for patients receiving tofacitinib plus MTX improved at week 52 in both the tofacitinib discontinuation group and the MTX discontinuation group. CDAI remission rates were 46.4% and 38.6% of patients, respectively (figure 1A, B). Patients who did not achieve clinical remission at week 52 did not move to the following (drug discontinuation) step and were treated with other csDMARDs or tsDMARDs. Two patients who achieved clinical remission in both arms refused withdrawal and dropped out of the study at this point. The other patients in clinical remission with tofacitinib plus MTX stopped tofacitinib or MTX according to the assignments at week 52 (figure 1). There were no differences in patient clinical characteristics except for RF and ACPA titers and positivity at discontinuation between the two groups (online supplemental table 1).

After the withdrawal of each drug, the proportion of patients with disease relapses increased with time in both the tofacitinib and MTX discontinuation groups. Out of patients who discontinued tofacitinib, 29.2% (7/24) of patients remained in remission (one patient experienced a period of low disease activity, but subsequently achieved remission again), and 41.7% (10/24) maintained low disease activity at week 104 in patients who achieved remission at week 52 (figure 1A). In contrast, of the patients who stopped taking MTX, 50.0% (10/20) of patients sustained in remission and 65.0% (13/20) maintained low disease activity (figure 1B). Notably, the loss of clinical remission occurred at a faster rate in the tofacitinib discontinuation group than in the MTX discontinuation group. When we compared the two groups, the CDAI remission and low disease activity rate were numerically higher in the MTX discontinuation group compared with the tofacitinib discontinuation group, but the difference was not statistically significant at week 104 (p=0.1576 and p=0.1228, respectively).

When considering the percentages of patients who achieved both clinical remission and successful discontinuation from baseline, the tofacitinib discontinuation group had 12.5% (7/56) of patients achieving this endpoint, whereas the MTX discontinuation group had 17.5% (10/57) of patients achieving this endpoint, and there were no statistical differences (p=0.4533 for remission and p=0.5135 for low disease activity) (figure 1A, B).

### Safety

Before discontinuation of tofacitinib or MTX at week 52, a total of 8 (out of 113) SAEs leading to withdrawal from the study occurred (online supplemental table 2). On the other hand, a total of 10 patients dropped out from this study before week 52 because of the lack of efficacy or economic burden (online supplemental table 2). After discontinuation of tofacitinib or MTX, no SAEs were recorded in both groups. However, 14 patients in the tofacitinib discontinue group and 7 patients in the MTX discontinue group dropped out from this study because of disease activity relapses as described above (figure 1A, B and online supplemental table 2).

### Predictive factor for achieving discontinuation of tofacitinib

**Figure 2:**
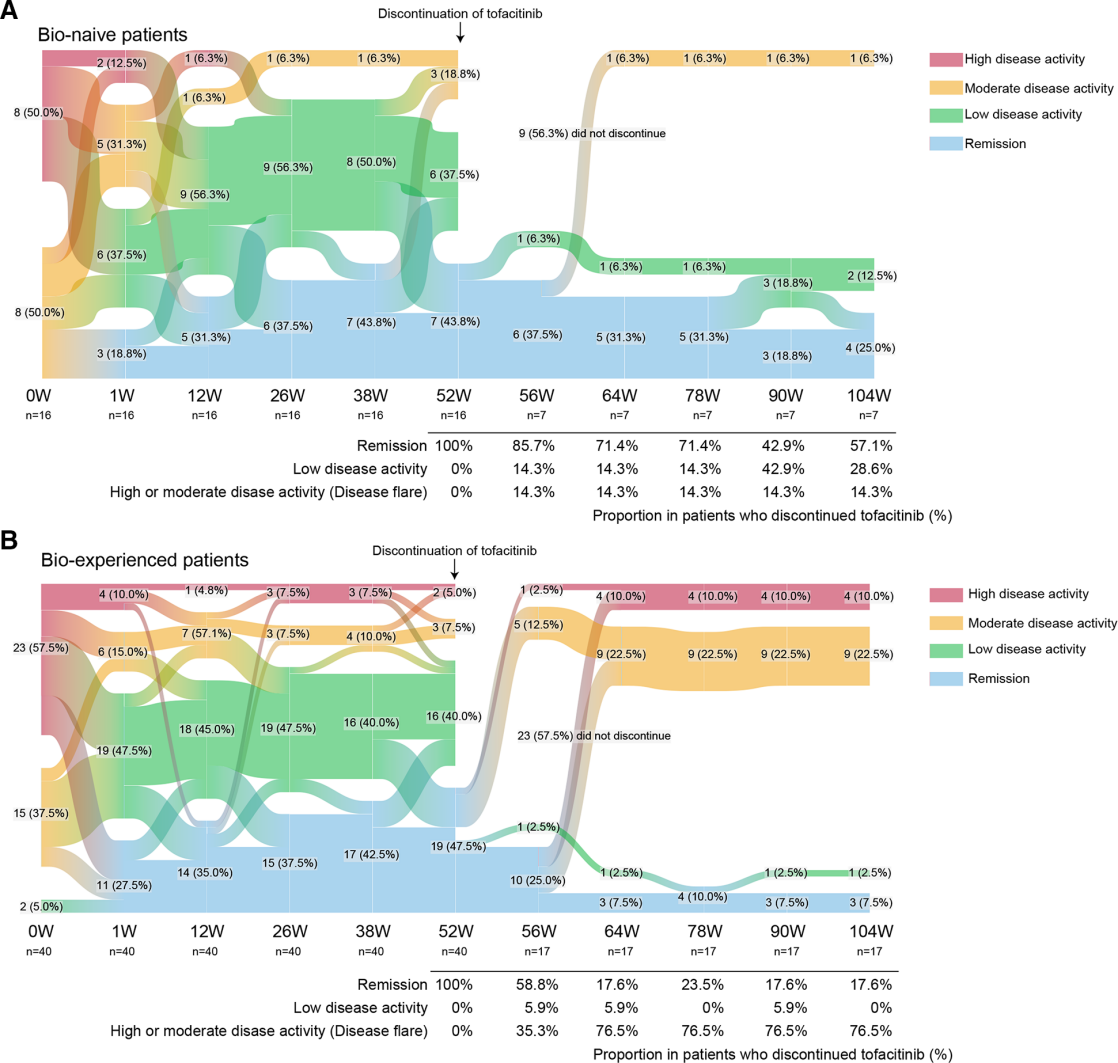
Sankey diagram describing proportion of patients per CDAI disease activity category over time in the subgroup. (A) Bio-naïve patients (n=16), (B) bio-experienced patients (n=21). Data are n (%). CDAI, Clinical Disease Activity Index.

As shown in figure 1, approximately 18% of the MTX-IR patients who were treated with tofacitinib plus MTX achieved clinical remission and subsequently sustained low disease activity with tofacitinib discontinuation. As an exploratory subgroup analysis, we, therefore, asked which patients could achieve both remission and successful drug discontinuation. We then divided the patients into two groups: those who were able to sustain low disease activity after drug discontinuation and those who were not. While the proportion of patients achieving clinical remission at week 52 was similar between groups, a greater proportion of bio-naïve patients maintained low disease activity with tofacitinib discontinuation at week 104, compared with the proportion of bio-experienced patients (online supplemental table 3). Considering the proportion from the baseline, 37.5% of bio-naive patients achieved both remission and sustained low disease activity after discontinuation of tofacitinib, with a sensitivity of 0.60 and specificity of 0.78 (figure 2A, B). In the meantime, out of the patients who discontinued tofacitinib, 57.1% (4/7) remained in remission (figure 2A).

In addition, the patients who had high titers of ACPA were less likely to achieve it, although these were not statistically significant (p=0.0569). These trends were not observed in the MTX discontinuation group, where patients who were able to maintain remission following MTX discontinuation were characterised by high matrix metalloproteinase-3 before starting tofacitinib (online supplemental table 3).

**Figure 3:**
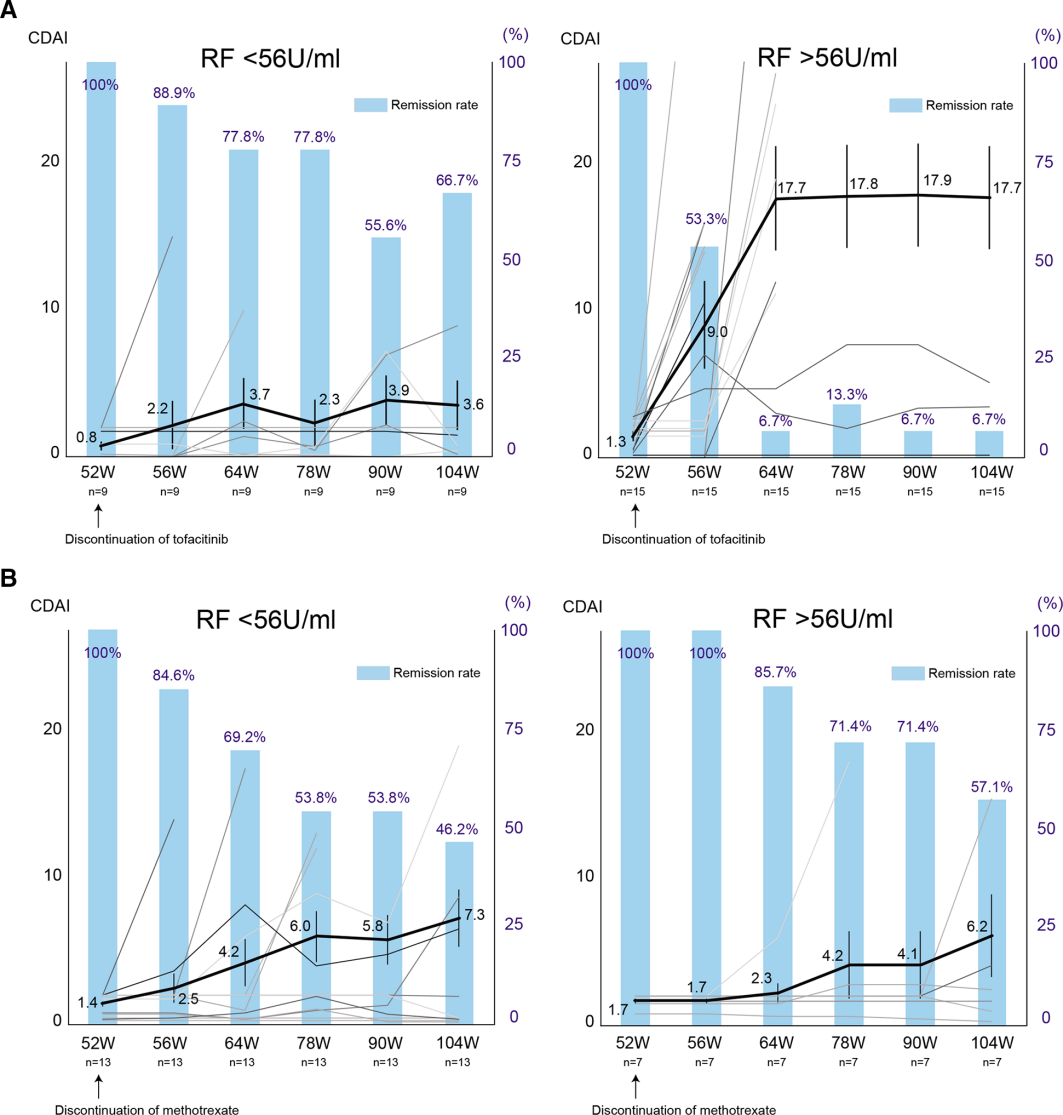
Serial changes in the CDAI and clinical remission according to CDAI after discontinuation of tofacitinib or methotrexate in the subgroup. Spaghetti plot (black lines: mean value, grey lines: the values of individual patients) and bar graph of remission rate are shown. (A) RF lower titre group (left) and higher titre group (right) in the tofacitinib discontinuation arm. (B) RF lower titre group (left) and higher titre group (right) in the methotrexate discontinuation arm. Data are mean (SEM) and %. CDAI, Clinical Disease Activity Index; RF, rheumatoid factor.

On the other hand, when comparing the patients’ backgrounds at week 52 (table 2), the patients who were able to discontinue tofacitinib without flares had lower RF (p=0.0404) and lower ACPA (p=0.05058). ROC curve analysis revealed the cut-off value of RF contributing to the sustained low disease activity after tofacitinib discontinuation as 56 U/mL (sensitivity 0.8, specificity 0.85). When the patients were divided into two groups according to the cut-off value, 66.7% of patients with low RF levels, including RF-negative patients (4 out of 9) with titers less than 15 U/mL, remained in remission at week 104, indicating a high probability of successful drug discontinuation (figure 3A). In contrast, only 6.7% of patients with higher RF sustained clinical remission after tofacitinib discontinuation. Of note, this trend was not observed in the MTX discontinuation group (figure 3B), and lower CDAI at the drug discontinuation was the main factor in the successful discontinuation of MTX (table 2).

**Table 2:** Key factors at the drug discontinuation of tofacitinib or methotrexate to achieve sustained low disease acitivity

Variables	Discontinuation of tofacitinib (n=24)			Discontinuation of methotrexate (n=20)		
	Sustained LDA	Non-sustained LDA	P value	Sustained LDA	Non-sustained LDA	P value
	n=10	n=14		n=13	n=7	
Age (years)	59.3 (12.4)	57.4 (11.2)	0·5383	57.5 (12.0)	52.4 (16.3)	0.6914
Gender, n (% female)	8 (80.0)	14 (100.0)	0.1630	12 (92.3)	4 (57.1)	0.1011
Disease duration (month)	52.9 (70.7)	139.3 (110.6)	0.0150	83.6 (85.4)	106.0 (71.9)	0.4509
Steinbrocker’s classification, %, I/II/III/IV	20/60/10/10	7/57/7/29	0.6144	23/54/8/15	29/29/13/29	0.7305
Prior use of biologics	4 (40.0%)	13 (92.9%)	0.0090	8 (61.5%)	4 (57.1%)	1.0000
MTX use at baseline, n (%)	10 (100.0%)	14 (100.0%)	1.0000	13 (100.0%)	7 (100.0%)	1.0000
Dose, mg/week	12.4±3.0	9.0±3.7	0.0243	12.0±3.7	14±2.3	0.2841
Glucocorticoid use at baseline, n (%)	0 (0.0%)	2 (14.3%)	0.4928	1 (7.7%)	0 (0.0%)	1.0000
Dose, mg/day		4.7 (0.3)	–	2.5		–
Tofacitinib dose /day, n (%)	5 mg; 0 (0.0), 10 mg; 10 (100.0)	5 mg; 1 (7.1), 10 mg; 10 (92.9)	1.0000	5 mg; 3 (23.1), 10 mg; 10 (76.9)	5 mg; 1 (14.3), 10 mg; 6 (85.7)	1.0000
28-Tender joint count	0.1 (0.3)	0.2 (0.4)	0.8160	0.0 (0.0)	1.1 (1.5)	0.0044
28-Swollen joint count	0.1 (0.3)	0.0 (0.0)	1.0000	0.1 (0.3)	0.3 (0.8)	0.6344
PGA, VAS 0–100 mm	12.3 (20.4)	10.1 (9.9)	0.4084	9.0 (7.0)	11.9 (9.2)	0.9682
EGA, VAS 0–100 mm	3.4 (9.1)	3.5 (5.2)	0.1206	3.4 (6.0)	4.7 (5.2)	0.6252
DAS28-ESR	2.1 (0.3)	2.3 (0.5)	0.2309	2.1 (0.4)	2.4 (0.6)	0.2039
CDAI	1.0 (1.5)	1.3 (0.9)	0.2615	1.3 (0.7)	2.0 (0.1)	0.0269
HAQ-DI	0.2 (0.2)	0.4 (0.4)	0.0878	0.6 (0.7)	0.5 (0.8)	0.5466
CRP (mg/dL)	0.1 (0.2)	0.1 (0.1)	0.7376	0.1 (0.1)	0.1 (0.1)	0.4477
ESR (mm/hour)	19.9 (16.2)	27.4 (19.6)	0.1851	16.5 (8.1)	16.6 (16.7)	0.3734
Rheumatoid factor (U/mL)	50.1 (60.3)	128.1 (144.6)	0.0404	46.3 (64.2)	72.6 (115.5)	0.8205
Rheumatoid factor positive, n (%)	7 (70.0)	12 (85.7)	0.3500	5 (38.5)	4 (57.1)	0.4231
Anti-CCP antibody (U/mL)	22.2 (33.0)	230.5 (547.2)	0.0508	250.4 (498.7)	82.1 (135.4)	0.2212
Anti-CCP antibody positive, n (%)	5 (50.0)	13 (92.9)	0.0168	8 (61.5)	4 (57.1)	0.8482
MMP-3 (ng/mL)	58.8 (28.4)	50.4 (31.4)	0.1602	45.2 (12.7)	47.3 (21.6)	0.8919

Data reported as mean (SD).

CCP, cyclic citrullinated peptide; CDAI, Clinical Disease Activity Index; CRP, C reactive protein; DAS28, Disease Activity Score 28 Joint; EGA, Evaluator’s Global Assessment; ESR, erythrocyte sedimentation rate; HAQ, Health Assessment Questionnaire; LDA, low disease acitivity; MMP-3, matrix metalloproteinase-3; MTX, methotrexate; PGA, Patient’s Global Assessment.

### Rescue by re-administration of tofacitinib or MTX

**Figure 4:**
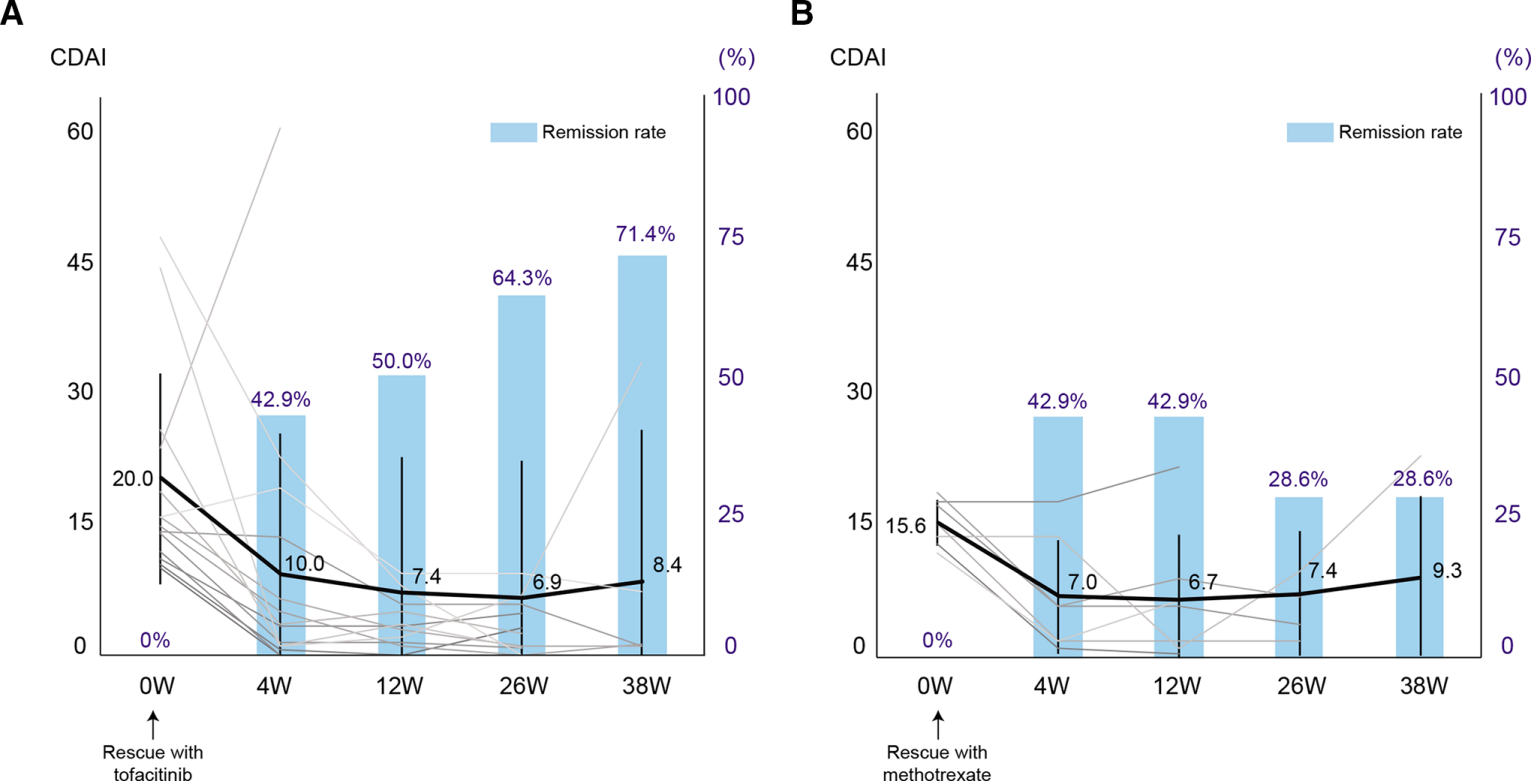
Serial changes in the CDAI and clinical remission according to CDAI after rescue by re-administration of tofacitinib or methotrexate. Spaghetti plot (black lines: mean value, grey lines: the values of individual patients) and bar graph of remission rate are shown in the tofacitinib discontinuation group (A) and in the methotrexate discontinuation group (B). Data are mean (SD) and %. CDAI, Clinical Disease Activity Index.

An important issue concerning drug discontinuation is whether re-administration of the drug following initial discontinuation can rescue flares of the disease activity. To address this issue, we investigated the clinical activity after re-administration of tofacitinib or MTX in patients with flares. These patients were re-administrated tofacitinib in the tofacitinib discontinuation group and re-administrated MTX in the MTX discontinuation group. In the tofacitinib discontinuation group, 58.3% (14 patients) relapsed with a mean CDAI of 20.1 at the time of flares, which improved to a mean CDAI of 8.4 with the resumption of tofacitinib (figure 4A). As a result, 71.4% of patients re-achieved clinical remission and 85.7% (71.4% plus 14.3% in low disease activity) re-achieved low disease activity (figure 4A). Meanwhile, the mean CDAI of those with disease flares in the MTX discontinuation group was 15.6, which was lower than that in the tofacitinib discontinuation group, and 28.6% of patients re-achieved remission (plus 42.9% of patients re-achieved low disease activity) after re-administration of MTX (figure 4B).

## Discussion

This study is an open-label, multicentre, randomised, prospective controlled trial for the withdrawal of tofacitinib. We note that there are three important clinical questions that this study addressed.

The first clinical question is the proportion of patients who could sustain clinical remission after tofacitinib discontinuation. In this study, 29.2% of patients sustained remission for 1 year after the withdrawal of tofacitinib. The percentage was not high. However, 70% of the patients who participated in this study had histories of using biologics. In this regard, 57.1% of the patients showed sustained clinical remission after withdrawal of tofacitinib when limited to bio-naive patients. This result’s comparison with the achievement of drug withdrawal for biologics is not straightforward, considering different patient characteristics.^20–25^ However, given that 58.3% of patients who discontinued tofacitinib relapsed in this study and a vast majority of the patients who continued tofacitinib did not exhibit disease flare in the previous observational study, it would be difficult to discontinue JAK inhibitors.^14^ On the other hand, MTX withdrawal may be more realistic, as only 35% of patients relapsed after MTX discontinuation. The 2022 updated EULAR treatment recommendation for RA stated that there was no significant difference in clinical outcomes between tapering a bDMARD or a csDMARD as the initial step.^26^ This study supports that tapering the csDMARD is preferable to tapering the tsDMARD in patients who have achieved remission. On the other hand, the ACR/EULAR international task force described that while either dose reduction or interval increase (also known as ‘spacing') is preferred, completely stopping treatment may not be advisable.^26^ Our findings endorse the dose reduction strategy suggested by the Task Force, and we anticipate more studies focused on tapering rather than discontinuing JAK inhibitors.

The second clinical question is about the treatment responsiveness in the patients who experienced a disease flare after drug withdrawal. In clinical practice, a gradual decrease in dosage is often preferred over drug withdrawal, and it is based on an empirical rule that it is less likely to result in disease flares than withdrawal.^11^ For example, regarding JAK inhibitors, 67% of patients could sustain low disease activity over a 2-year period after the dose of baricitinib was reduced from 4 mg to 2 mg.^27^ However, drug withdrawal still could be an option if the resumption of the drug results in an improvement in disease activity. Previous studies on the withdrawal of a tumor necrosis factor (TNF) inhibitor showed that resuming the TNF inhibitor could induce clinical remission or low disease activity in more than 80% of patients who experienced relapse after withdrawal.^28^ The present study also showed that re-administration of tofacitinib after disease flare allowed 85.7% of patients to achieve low disease activity and 71.4% of patients to achieve remission, suggesting that the withdrawal of tofacitinib is unlikely to result in the acquisition of treatment-resistance. In other words, our results align with previous research on discontinuing bDMARDs after achieving clinical remission, where most patients (though not all) who experienced a flare were able to achieve a favourable disease state after reintroduction of the medication.

In addition to these results, we found that no SAEs were observed after discontinuation of tofacitinib or MTX. Presumably, the decrease in adverse events could be the case since drug withdrawal theoretically eliminates the incidence of side effects. However, the patients were already treated for a year with tofacitinib before discontinuation. Therefore, a natural selection of patients who were less prone to SAE in the second year could already have occurred. Moreover, a larger number of patients are generally required to study adverse events rather than to investigate clinical efficiency, and thus, a follow-up study is necessary to explore the safety issues.

We also made an observation from post hoc analysis. In our study, lower serum titers of RF and ACPA were found to be the factors for the successful discontinuation of tofacitinib. Similar results were obtained in our previous studies.^14^ However, the prediction cannot be universally applied due to the study’s lack of statistical power.

There are some limitations in this study. We estimated the sample size based on the previous papers and our preliminary data,^14 29 30^ however, there are underpowered enrolled patients to detect the difference regarding primary outcomes since there was not enough evidence to estimate an accurate proportion for successful MTX/tofacitinib discontinuation. In fact, there was a numerically different in successful discontinuation rate (29.2% for tofacitinib discontinuation vs 50.0% for MTX discontinuation) in patients who achieved remission on MTX plus tofacitinib, but not statistically different. Additionally, the utilisation of simple randomisation instead of constrained randomisation may have resulted in unintended bias. The patients were randomised before starting tofacitinib, which could risk having discrepancies in the characteristics of patients at the time of discontinuation. As a result, there was an imbalance in RF/ACPA levels and positivity, making it difficult to interpret the results. Second, we could not make double placebo pills to keep blinded during the discontinuation periods. Therefore, this study is open-label study during the discontinuation of tofacitinib or MTX, which could cause a nocebo effect and affect the clinical response. Finally, our study did not include the third arm, which consisted of the patients who continued to take tofacitinib and MTX. However, our preliminary observation study for this paper showed that the vast majority of patients who continued to tofacitinib maintained the same disease activity for 52 weeks.^14^ In addition, this study could not compare the gradual decrease and withdrawal of drugs, although both are essential options.

This study investigated sustained remission following the cessation of tofacitinib, which revealed that the discontinuation of JAK inhibitors is not the universal best option, and that MTX discontinuation is a more practical option. It is crucial for the successful tofacitinib withdrawal to carefully select patients.

## Data Availability

All data relevant to the study are included in the article or uploaded as supplementary information.
