# Supplementary material for: Sustained remission following the discontinuation of tofacitinib in patients with rheumatoid arthritis (XANADU study): an open-label randomised study
Source: RMD Open. 2023 Apr 25;9(2):e003029. doi: 10.1136/rmdopen-2023-003029 (PMC10152036; doi:10.1136/rmdopen-2023-003029)
Supplement: Supplementary data [file rmdopen-2023-003029supp001.pdf]

S Kubo et al. Xanadu study supplemental tables 1

Supplemental Table S1. Baseline clinical characteristics at discontinuation

Variables	Discontinuation of tofacitinib (n=24)	Discontinuation of methotrexate (n=20)	p value
Disease duration (month)	103.5 (79.8)	115.3 (103.8)	0.9061
Prior use of biologics	17 (70.8%)	12 (60.0%)	0.5316
28-tender joint count	0.4 (1.0)	0.2 (0.4)	0.6733
28-swollen joint count	0.2 (0.5)	0.0 (0.2)	0.4341
PGA, VAS 0-100 mm	10.0 (7.7)	11.0 (14.8)	0.4349
EGA, VAS 0-100 mm	3.9 (5.7)	3.5 (6.9)	0.5693
DAS28-ESR	2.2 (0.5)	2.2 (0.5)	0.9812
CDAI	1.5 (0.7)	1.2 (1.2)	0.1002
HAQ-DI	0.6 (0.7)	0.3 (0.4)	0.2866
CRP (mg/dl)	0.1 (0.1)	0.1 (0.1)	0.7135
ESR (mm/hr)	16.6 (11.4)	24.3 (18.3)	0.1249
Rheumatoid factor (U/ml)	56.0 (84.5)	95.6 (121.6)	0.0375
Positive (%)	19 (79.2%)	9 (45.0%)	0.0190
Anti-CCP antibody (U/ml)	185.0 (399.9)	149.0 (433.7)	0.9686
Positive (%)	18 (75.0%)	12 (60.0%)	0.2875
MMP-3 (ng/ml)	46.0 (16.0)	53.7 (29.9)	0.6311

Data reported as mean (SD). PGA, patient’s global assessment; EGA, evaluator’s global assessment; DAS28, disease activity score 28 joint; ESR, erythrocyte sedimentation rate; CDAI, clinical disease activity index; HAQ, health assessment questionnaire; CRP, C reactive protein; CCP, cyclic citrullinated peptide.

S Kubo et al. Xanadu study supplemental tables 2

Supplemental Table S2. The reason of withdrawal from Xanadu study

	Discontinuation of tofacitinib (n=56)	Discontinuation of methotrexate (n=57)	p value
Until week 52			
Serious adverse events	2 (3.6%)	6 (10.5%)	0.1495
	Shingles<br>Appetite loss	Malignancy (lymphoma, Pancreatic cancer)<br>Oral aphtha<br>General fatigue<br>Appetite loss<br>Eruption	
Lack of efficacy	3 (5.4%)	5 (8.8%)	0.4769
Economic burden	2 (3.6%)	0 (0.0%)	0.1500
Between week 52 to week 104			
Serious adverse events	0 (0.0%)	0 (0.0%)	-
Disease flare	14 (25.0%)	7 (12.3%)	0.1228

S Kubo et al. Xanadu study supplemental tables 3

Supplemental Table 3 Key factors at baseline to achieve clinical remission and the following sustained LDA after the discontinuation of tofacitinib or methotrexate.

	Discontinuation of tofacitinib (n=56)				Discontinuation of methotrexate (n=57)		
Variables	Sustained LDA	Others	p value		Sustained LDA	Others	p value
	n=10	n=46			n=13	n=44	
Age (years)	59.3 (12.4)	60.3 (11.7)	0.8472		57.5 (12.0)	56.4 (14.8)	0.8715
Gender, n (% female)	8 (80.0%)	40 (87.0%)	0.5688		12 (92.3%)	35 (79.6%)	0.2878
Disease duration (month)	52.9 (70.7)	106.2 (103.1)	0.0671		83.6 (85.4)	119 (107.6)	0.2092
Steinbrocker's classification, %, I/II/III/IV	20/60/10/10	17/50/13/20	0.8798		23/54/8/15	25/36/18/21	0.6575
Prior use of biologics	**6 (60.0%)**	**36 (78.3%)**	**0.0152**		8 (61.5%)	28 (63.6%)	0.8904
MTX use at baseline, n (%)	10 (100.0%)	46 (100.0%)	1		13 (100.0%)	44 (100.0%)	1
dose, mg/w	12.4 (3.0)	11.2 (4.2)	0.4404		12.0 (3.7)	12.5 (3.5)	0.7545
Glucocorticoid use at baseline, n (%)	0 (0.0%)	6 (13.0%)	0.2268		1 (7.7%)	3 (6.8%)	0.9137
dose, mg/day	-	5.0 (3.6-5.0)	-		2.5	5.0 (5.0-7.5)	-
Tofacitinib dose, n (%)	5mg; 0 (0.0%)<br>10mg; 10 (100.0%)	5mg; 2 (4.4%)<br>10mg; 44 (95.7%)	0.5019		5mg; 3 (23.1%)<br>10mg; 10 (76.9%)	5mg; 3 (6.8%)<br>10mg; 41 (93.2%)	0.0933
28-tender joint count	7.1 (6.2)	8.7 (6.0)	0.4212		7.6 (6.2)	9.1 (5.8)	0.375
28-swollen joint count	8.4 (3.8)	8.2 (4.9)	0.7394		6.7 (4.3)	6.7 (4.3)	0.6398
PGA, VAS 0-100 mm	61.4 (23.3)	54.4 (25.7)	0.4223		46.8 (17.6)	49.3 (24.0)	0.7826
EGA, VAS 0-100 mm	51.3 (18.4)	45.2 (22.5)	0.2655		42 (17.1)	44.6 (22.4)	0.8194
DAS28-ESR	5.5 (1.2)	5.5 (1.3)	0.8473		5.4 (1.2)	5.2 (1.2)	0.6419
CDAI	27.7 (11.1)	27.4 (12.6)	0.881		24.6 (11.9)	25.8 (11.1)	0.6209
HAQ-DI	1.0 (0.8)	1.3 (0.8)	0.2428		1.3 (0.7)	1.1 (0.8)	0.4867
CRP (mg/dl)	2.9 (3.2)	1.9 (3.3)	0.2523		2.4 (4.2)	1.7 (2.7)	0.2867
ESR (mm/hr)	52.7 (39.9)	43.9 (31.1)	0.6151		47.8 (28.0)	36.5 (31.0)	0.1232
Rheumatoid factor (U/ml)	93.2 (102.5)	152.5 (179.2)	0.3095		75.2 (101.0	202.0 (397.6)	0.6618
Positive (%)	8 (80.0%)	42 (91.3%)	0.2949		8 (61.5%)	28 (63.6%)	0.8904
Anti-CCP antibody (U/ml)	**94.6 (162.4)**	**206.7 (310.8)**	**0.0569**		226.1 (440.3)	165.3 (406.3)	0.1707
Positive (%)	**6 (60.0%)**	**43 (93.5%)**	**0.0037**		10 (76.9%)	29 (65.9%)	0.4529
MMP-3 (ng/ml)	412.4 (609.3)	230.8 (312.5)	0.2901		344.1 (405.6)	155.1 (201.4)	**0.0251**

Data reported as mean (SD). MTX, methotrexate; PGA, patient’s global assessment; EGA, evaluator’s global assessment; DAS28, disease activity score 28 joint; ESR, erythrocyte sedimentation rate; CDAI, clinical disease activity index; HAQ, health assessment questionnaire; CRP, C reactive protein; CCP, cyclic citrullinated peptide； MMP-3, matrix metalloproteinase-3.
