# Supplementary figures and images for: Sustained remission following the discontinuation of tofacitinib in patients with rheumatoid arthritis (XANADU study): an open-label randomised study
Source: RMD Open. 2023 Apr 25;9(2):e003029. doi: 10.1136/rmdopen-2023-003029 (PMC10152036; doi:10.1136/rmdopen-2023-003029)

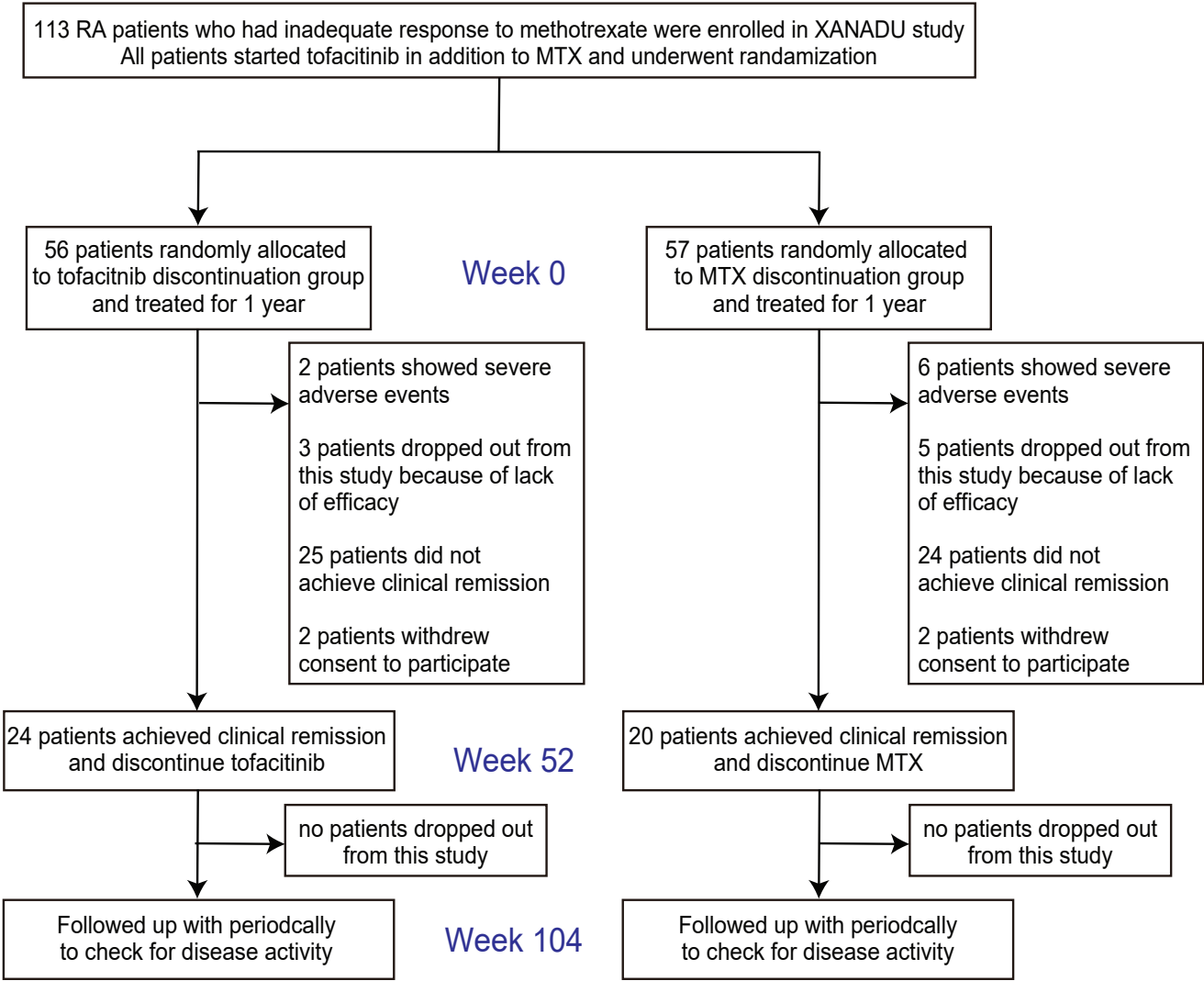

Supplement: Supplementary data [file rmdopen-2023-003029supp002.pdf]
